# Diseases of the Respiratory Organs; Acute and Chronic

**Published:** 1901-09

**Authors:** 


DISEASES OF THE RESPIRATORY ORGANS; ACUTE AND CHRONIC. Arranged in ten parts, and interleaved for supplemental notes. By Wm. F. Waugh, A. M., M. D., Professor of Practice and Clinical Medicine, Illinois Medical College, etc. The Clinic Publishing Co., Chicago. 1901. $1.00.

Dr. Waugh is well known to the medical profession as a clear, concise and forceful writer, exact in his statements and generally correct as to his position. The book will be found to be a very complete manual for frequent reference, and will be especially beneficial and instructive to the younger members and recent graduates, whose reading has not been sufficiently extensive to cover all the results here summarized by this talented author.

